# The cognitive neuropsychological phenotype of carriers of the *FMR1* premutation

**DOI:** 10.1186/1866-1955-6-28

**Published:** 2014-07-30

**Authors:** Jim Grigsby, Kim Cornish, Darren Hocking, Claudine Kraan, John M Olichney, Susan M Rivera, Andrea Schneider, Stephanie Sherman, Jun Yi Wang, Jin-Chen Yang

**Affiliations:** 1 Department of Psychology, University of Colorado Denver, Denver, CO, USA; 2 Department of Medicine; Division of Health Care Policy and Research, University of Colorado School of Medicine, Aurora, CO, USA; 3 School of Psychology & Psychiatry; Faculty of Medicine, Nursing and Health Sciences, Monash University, Melbourne, Victoria, Australia; 4 Olga Tennison Autism Research Centre, School of Psychological Science, La Trobe University, Melbourne, Victoria, Australia; 5 Center for Mind and Brain, University of California, Davis, CA, USA; 6 Department of Neurology, University of California, Davis, Sacramento, CA, USA; 7 Department of Psychology, University of California-Davis, Sacramento, CA, USA; 8 MIND Institute, University of California-Davis Medical Center, Sacramento, CA, USA; 9 Department of Human Genetics, Emory University, Atlanta, GA, USA; 10 Department of Pediatrics, University of California, Davis, Sacramento, CA, USA

**Keywords:** Fragile X-associated tremor/ataxia syndrome, FXTAS, FMR1, Fragile X, Executive function, Cognition disorders, Fragile X premutation

## Abstract

The fragile X-associated tremor/ataxia syndrome (FXTAS) is a late-onset neurodegenerative disorder affecting a subset of carriers of the *FMR1* (fragile X mental retardation 1) premutation. Penetrance and expression appear to be significantly higher in males than females. Although the most obvious aspect of the phenotype is the movement disorder that gives FXTAS its name, the disorder is also accompanied by progressive cognitive impairment. In this review, we address the cognitive neuropsychological and neurophysiological phenotype for males and females with FXTAS, and for male and female unaffected carriers. Despite differences in penetrance and expression, the cognitive features of the disorder appear similar for both genders, with impairment of executive functioning, working memory, and information processing the most prominent. Deficits in these functional systems may be largely responsible for impairment on other measures, including tests of general intelligence and declarative learning. FXTAS is to a large extent a white matter disease, and the cognitive phenotypes observed are consistent with what some have described as white matter dementia, in contrast to the impaired cortical functioning more characteristic of Alzheimer’s disease and related disorders. Although some degree of impaired executive functioning appears to be ubiquitous among persons with FXTAS, the data suggest that only a subset of unaffected carriers of the premutation - both female and male - demonstrate such deficits, which typically are mild. The best-studied phenotype is that of males with FXTAS. The manifestations of cognitive impairment among asymptomatic male carriers, and among women with and without FXTAS, are less well understood, but have come under increased scrutiny.

## 

The fragile X-associated tremor/ataxia syndrome (FXTAS) is a late-onset neurodegenerative disorder associated with trinucleotide (CGG) repeat expansions of the *FMR1* (fragile X mental retardation 1) gene. The most obvious signs and symptoms of FXTAS include action tremor, ataxia, parkinsonism, and peripheral neuropathy, but circumscribed aspects of cognition are impaired as well. FXTAS affects persons who have CGG expansions in the *premutation* range (that is, 55–200 CGG repeats), as opposed to fragile X syndrome (FXS), which is associated with the full mutation (over 200 CGG repeats) at *FMR1*. FXTAS and FXS have very different phenotypes, the latter involving an early childhood-onset intellectual development disorder, often with autistic features, while FXTAS affects persons who previously were cognitively normal, in the sixth or seventh decade of life.

The basic cognitive phenotype of FXTAS is relatively well understood. However, at the time of the initial identification of FXTAS, it was thought that women who carry the premutation might have little susceptibility to the disorder, perhaps because of the presence of a normal allele among female heterozygotes. Penetrance, and possibly expression, is significantly higher in males than females, and because men with FXTAS were more readily identified than females with FXTAS, they have been more extensively studied, and more is known about the nature, severity, and course of cognitive impairment among men. The objective of this paper is to review the cognitive neuropsychological phenotype of carriers of the fragile X (FX) premutation, both those who have FXTAS, and those who are clinically unaffected.

## Review

### Cognitive neuropsychological phenotype among males with FXTAS

Since the first case reports [1], it has been apparent that FXTAS is associated with a progressive decline in cognitive functioning. Although deficits in several aspects of cognition have been reported [2-4], the evidence appears to support the idea that the primary disorder is a disturbance of executive functioning (EF) [5]. The executive functions represent an important subset of cognitive abilities. In essence, EF is the capacity for the autonomous regulation of one’s own behavior and attention. Fuster [6,7] maintains that EF represents the temporal organization of behavior, allowing one to initiate purposeful behavior, and to inhibit irrelevant or inappropriate activity.

Early studies of FXTAS reported deficits in EF, working memory, speed/capacity of information processing and learning [2,3,8]. The first systematic study of cognition essentially replicated the early results, and showed that males with FXTAS ranged from high average through severely impaired in these areas of functioning, the group mean representing moderate impairment [4]. FXTAS patients performed significantly worse on several EF measures than did a sample of controls.

General cognitive functioning among FXTAS patients is typically impaired [2-4], although intelligence quotient (IQ) scores are in the normal range. In the largest study of men with FXTAS to date, mean Wechsler Adult Intelligence Scale-III (WAIS-III) verbal and performance (nonverbal) IQ scores were 106.6 and 97.7, respectively [4]. Lower performance IQs may be a function of the greater dependence of the nonverbal scale on processing speed, working memory, and EF [5]. On the Mini Mental State Exam [9], a measure of general mental status, the difference in mean scores of FXTAS patients and controls was statistically, but not clinically, significant (27.6 vs 29.2). Nevertheless, a subset of individuals with FXTAS - most with advanced disease - demonstrates frank dementia [10,11]. Performance on measures of declarative memory is impaired, likely reflecting the effects of executive dysfunction (that is, problems with active learning and active retrieval) rather than a primary memory disorder.

Neither expressive nor receptive language appears to be affected in FXTAS. Repetition, naming, reading, comprehension of complex material, and following spoken commands differed little across groups. Performance on most visuospatial measures was unimpaired, although men with FXTAS performed more poorly than controls on the WAIS-III Block Design subtest - probably in association with reduced processing speed and deficient organizational ability [4,5].

In a study of functional status (basic physical functioning, activities of daily living, and instrumental activities of daily living), men with FXTAS, but not unaffected carriers, were impaired in all three functional areas. This effect appeared to be mediated by both executive and motor functioning [12].

It is not possible to localize the cognitive deficits of FXTAS to a specific brain region. Although the event-related brain potential (ERP) studies of Yang and colleagues [13,14] may implicate prefrontal cortex, EF is a widely distributed functional system that also may be affected by lesions of other areas, in particular the cerebellum [15,16]. To a large extent, FXTAS is a white matter disease [17-19], and the cognitive phenotype observed among men with FXTAS is similar to what has been called white matter dementia [16,20,21], in which the extent of executive deficits is correlated with the burden of white matter abnormalities distributed throughout the brain [22].

### Cognitive neuropsychological phenotype among females with FXTAS

The manifestation of neuropsychological signs and symptoms in female carriers can be diverse with low penetrance of FXTAS and milder phenotypes than those observed in males. Based on current diagnostic criteria, estimates suggest that as few as 8 to 16 % of female carriers will develop FXTAS [23,24]. Moreover, in comparison with males, the onset of FXTAS for females tends to occur later in life, and is anatomically less severe, with a lower penetrance of middle cerebellar peduncle and pontine white matter hyperintensities, less brain atrophy, and less severe white matter disease overall [25,26]. This milder phenotype is thought to stem from protective mechanisms associated with X-inactivation and hormonal differences [27-29]. However, this notion does not have full support [23].

Although studies of FXTAS among males suggest that the presenting cognitive profile is predominantly one of executive dysfunction, there is a dearth of research systematically examining the cognitive profile in females with FXTAS. Indeed, existing studies have been inconsistent in demonstrating interrelationships between neuropsychological profiles, imaging correlates, and standardized clinical measures [25,30-32]. Recent neuropsychological and neurophysiological studies have shown that females with FXTAS present with executive dysfunction as the primary cognitive impairment, similar to the male cognitive profile [32,33]. Most significantly, a neuropathological case study series reported dementia in 4 of 8 autopsied female carriers, in conjunction with an increased number of intranuclear inclusions, suggesting that dementia may be more prevalent than previously thought in women with FXTAS [34].

With regard to psychological symptoms in females with FXTAS, neuroimaging studies have shown an association between hippocampal abnormalities and anxiety in unaffected carriers [35]. Although these results suggest that females show mild early-onset neuropsychological phenotypes irrespective of FXTAS, further research is needed to understand the synergistic effects of age, psychological vulnerability, and risk factors for FXTAS. Indeed, given that prevalence rates for probable attention deficit hyperactivity disorder (4.5 to 25%), anxiety (20.9 to 30%) and depression (14.03 to 47.3%) far exceed the risk of FXTAS (8 to 16%) in women [23,24,36-40], the molecular and environmental factors contributing to gender-divergent trajectories are yet to be fully characterized.

### Neurophysiology of cognition in FXTAS

Much of what is known of the cognitive electrophysiology of FXTAS patients has been found using the ERP technique. Cognitive ERPs, primarily representing the summated activity of post-synaptic potentials, offer an excellent non-invasive measure for characterizing different stages of cognitive processing due to their millisecond-level temporal resolution [41].

While performing an auditory “oddball” task requiring dual-response, a predominantly male group of participants with FXTAS had longer N100 latency and smaller P200 amplitude than normal controls, indicating some impairment of early-stage attention [13]. Furthermore, these individuals showed decreased amplitude and prolonged latency of P300, a component believed to reflect late-stage “controlled” attention and working memory updating. P300 abnormalities in these FXTAS patients were observed over fronto-central and parietal scalp, and the frontal P300 measures correlated with executive function test scores, working memory, *FMR1* mRNA level, and CGG repeat length [13]. The frontal P300 impairments have been replicated in a second study including 29 female FXTAS patients, with correlations also obtained between P300 measures and executive function, working memory, and information processing speed [32]. Therefore, it has been suggested [13,32] that abnormal fronto-parietal attentional network dynamics may play an important role in executive dysfunction, the primary cognitive impairment in FXTAS [5].

Verbal memory and language processes in FXTAS also have been probed via an ERP word repetition paradigm/category judgment task, in which semantically congruous (50%) and incongruous words are repeated after an interval of between 10 and 140 seconds. This word repetition paradigm was employed in the first published ERP study of FXTAS [42], in which it was demonstrated that FXTAS patients had a significantly reduced N400 word repetition effect (new-old incongruous words), an electrophysiological marker of semantic processing load and implicit memory previously shown to be sensitive to early Alzheimer’s disease (AD) [43,44]. Unlike persons with AD, however, FXTAS patients displayed a preserved P600 word repetition effect (new-old congruous words) that has been linked to episodic/declarative memory. Yang and colleagues [45] examined a group of FXTAS females and found a relatively normal N400 repetition effect, but decreased N400 congruity effect (incongruous-congruous new words), suggesting abnormal semantic activation and/or semantic network disorganization, which they attributed to executive dysfunction.

Electrophysiology has also been used to assess the effectiveness of potential pharmacologic treatments for FXTAS. Because the N400 measures are sensitive to both FXTAS and glutamatergic signaling [46], the method was utilized to evaluate the treatment effects of memantine (an uncompetitive N-methyl-d-aspartate receptor antagonist approved for treatment of AD) in a substudy of the first placebo-controlled, double-blind, randomized clinical trial in FXTAS [47,48]. Results indicated a tendency toward larger N400 repetition effect amplitude after 12 months on memantine, and patients who had increased N400 repetition effects showed the largest improvements in cued-recall of the experimental verbal stimuli. Although the main memantine trial in FXTAS showed no significant treatment effects on the primary outcome measures of executive function and intention tremor [48], the ERP results suggest the need for further studies of memantine and/or other therapeutic agents for this neurodegenerative disorder, which currently has no proven therapies.

There is a paucity of other published studies on the central neurophysiology of FXTAS, with a few case report studies showing abnormal electroencephalogram (for example, [49]). One noteworthy study of people with FXTAS found decreased auditory prepulse inhibition [50], an electromyography index of sensorimotor gating also affected in FXS. Thus, abnormalities of the frontal P300, N400 brain potentials, and prepulse inhibition should all be considered putative electrophysiological trait markers of the *FMR1* premutation.

### Cognition among unaffected male premutation carriers

The cognitive phenotype of unaffected male carriers of the *FMR1* premutation, although not as extensively researched as that of males with FXTAS, is relatively well understood. There is general agreement that some males demonstrate a progressive age-related decline in executive function skills that is greater than that displayed by males with a normal allele, matched on age and IQ. In a series of published papers, Cornish and colleagues have isolated a subtle age-related decline of inhibitory control (as measured by the Haylings Task) and working memory (as measured by the Letter-Number sequencing task [51-53]) that begins in early adulthood and continues across the lifespan. Interestingly, males with more than 100 CGG repeats may be especially at risk for cognitive difficulties [51]. Grigsby and colleagues [4] reported a similar profile of difficulties in executive functioning, with unaffected carriers performing worse than normal-allele controls on a composite measure of EF, and on immediate and delayed recall on the Logical Memory subtest of the Wechsler Memory Scale-III [54].

In addition, recent functional magnetic resonance imaging (fMRI) data indicate changes in prefrontal activity in premutation carriers, irrespective of FXTAS diagnosis, during performance of a working memory task [55]. These preliminary findings provide the first evidence to suggest the involvement of specific brain regions associated with neural networks mediating EF among persons in the premutation range, with or without FXTAS. However, several studies have failed to find differences in cognitive performance between non-affected male carriers and typically developing males [56,57]. One possibility is that relatively small samples sizes and variability in age range and CGG repeat distribution across studies may explain some of the discrepant findings.

To address this concern, Hunter and colleagues [58] combined their samples to form a multi-center database with 100 asymptomatic premutation males and 216 non-carriers. In line with previous reports by Cornish and Grigsby [4,5,51], analyses across shared measures of inhibition and working memory detected very subtle differences (which were of minimal clinical significance) on a measure of response inhibition (Stroop task), but on no other cognitive measures. Taken together, current findings provide evidence of a subtle, suboptimal level of cognitive performance among a subset of unaffected carriers, involving mild deficits in inhibition and working memory among a subset of male carriers, results which are relatively consistent with earlier research [59-61].

### Cognition among unaffected female premutation carriers

Although the presence of a neuropsychological phenotype in unaffected female premutation carriers is controversial [56,57], recent studies have shown that a subset of young adult female carriers exhibit subtle deficits in executive function and inhibitory control [33,37,62], visuospatial processing [63,64], mathematical reasoning [65,66], and tasks dependent on processing speed and working memory [32]. It has been argued that targeted, domain-specific measures may more sensitively identify subtle neurocognitive deficits than gross neuropsychological tests [67]. Indeed, more targeted measures have identified enhanced psychomotor ability [68,69], motor programming deficits [70], increased balance and memory problems [70,71], and age- and CGG-dependent changes in the attentional demands of postural control [72]. Collectively, these findings point to early at-risk phenotypic features suggesting cerebellar dysfunction in a subgroup of female premutation carriers; however, prospective longitudinal studies will be needed to determine who will eventually develop cognitive impairment and neurodegenerative decline.

With regard to the neuropsychiatric phenotype, it has been shown that a subgroup of women who carry the *FMR1* premutation, but who do not have FXTAS, may also be more vulnerable to elevated symptoms of anxiety and depression compared to the general population [40,73,74]. At least in part this may be the result of genetic variation (for example, CRHR1 polymorphisms and release of cortisol) moderating the stress of raising a child with FXS [75,76]. However, one recent study has shown that poorer inhibitory control and working memory are associated with elevated social anxiety, depression, and attention deficit hyperactivity disorder (predominantly inattentive symptoms) among women with the *FMR1* premutation [37].

These data are novel in that they indicate early changes within frontally-mediated networks that may increase the risk of neuropsychiatric dysfunction. However, evidence for a contribution by other factors, such as expanded CGG repeats, and reproductive history in depressive female carriers, and anxiety symptoms in female carriers, has been inconsistent [37,75,76]. A recent study using more detailed clinical interviews from the Diagnostic and Statistical Manual of Mental Disorders, Fourth Edition has provided evidence that comorbid depressive and anxiety disorders are associated with significantly expanded CGG repeats, but not with reproductive or menstrual history [77]. Thus, a complex set of factors may contribute to executive and neuropsychiatric dysfunction in female carriers, involving genetic and epigenetic factors, caregiver burden, hormonal changes, and hypothalamic-pituitary-adrenal axis function. The precise relationships among these factors remain unclear.

### Neuroimaging and cognition

The *FMR1* gene and its protein product, fragile X mental retardation protein (FMRP), are expressed in brain areas important for high-level cognitive functioning [78,79]. Consequently, both subtle FMRP deficit and excessive *FMR1* mRNA may affect the brain during development and aging in the *FMR1* premutation, which ultimately lead to the cognitive phenotype in FX premutation carriers. Neuroimaging is a useful tool for non-invasive investigations of the impact of the *FMR1* premutation on the brain and neural substrates underlying the cognitive deficits. Two main imaging modalities provide complementary insights into the mechanisms: functional MRI, which examines brain activation during cognitive tasks, and structural MRI, which provides a quantification of volume, morphometry, and white matter structural integrity/connectivity.

Consistent with the FMRP expression profile in the brain [80], both functional and structural MRI studies have shown a dose–response of *FMR1* gene expression on brain regions important for working memory. In a functional MRI study examining verbal working memory in male and female premutation carriers [55], reduced activation was observed in the right inferior frontal cortex and left premotor cortex in both asymptomatic premutation carriers and carriers with FXTAS. Reduced activation was found in right premotor/inferior frontal cortex in the FXTAS group. The reduced activation in the right inferior frontal cortex exhibited a significant correlation with high lymphocytic *FMR1* mRNA levels. In a structural MRI study [80] of male premutation carriers, while individuals with FXTAS showed diffuse gray matter loss most prominent in areas important for working memory (including prefrontal cortex, anterior cingulate cortex, and cerebellum) only the anterior cerebellar vermis and hemisphere displayed volume loss in those premutation carriers without FXTAS. Importantly, the reduced gray matter density of the inferior frontal and anterior cingulate cortices correlated significantly with low working memory performance, and the reduced gray matter density in the dorsomedial frontal cortex correlated with long CGG repeat length in premutation carriers.

Functional MRI studies have also revealed differences in brain activation in young adult premutation carriers asymptomatic for FXTAS, even where subtle differences in cognition cannot be measured by standard behavioral techniques. In a study of delayed recall memory, premutation carriers showed no deficits (relative to control participants) in ability to recall pairings on an associative memory task a day later, but were found to exhibit reduced left hippocampal activation, and activity in the left hippocampus was also negatively correlated with both *FMR1* mRNA level and psychiatric symptomatology [81].

Likewise, in a study examining magnitude estimation processing, young asymptomatic adult carriers showed significantly attenuated bilateral intraparietal sulcus (IPS) activity that is commonly associated with the numerical distance effect (that is, greater effort/brain activation when the two displays to be compared are closer in numerosity), despite comparable behavioral task performance with the control group [82]. While both elevated *FMR1* mRNA and increased CGG repeat expansion were associated with reduced IPS activation, the results of multiple regression suggested that increased CGG repeat size was the primary contributor to aberrant IPS function.

### Molecular mechanisms of FXTAS

Although the data suggest that FXTAS may result from a toxic gain of function associated with excessive levels of *FMR1* mRNA, the mechanism, and variables that influence it, remain unclear [83]. Although CGG repeat size in the premutation range is a diagnostic indicator and genetic marker for those who may develop FXTAS, repeat length predicts only a portion of risk and severity of FXTAS [84,85], and there are other yet unidentified factors involved. Repeat length does not elucidate the mechanism of cellular or central nervous system pathology, or the relationship of cellular pathology to clinical disease.

### Treatment of FXTAS

There are as yet no effective therapies for the treatment of FXTAS. As noted previously, one 12-month randomized controlled trial of memantine showed no difference from placebo on measures of tremor severity and executive functioning [48]. The limited literature is based largely on case studies (for example, [86]) and treatment has largely been directed toward symptom amelioration, with inconsistent results [87,88].

## Conclusion

The cognitive phenotype of FXTAS for both sexes appears to be characterized by a progressive impairment of executive functioning, working memory, and speed of information processing. This is characteristic of the pattern observed in fronto-cerebellar and white matter disease. The degree of impairment may become severe, and in advanced stages people with FXTAS may have very significant cognitive problems, even demonstrating problems ordinarily associated with cortical disorders. The dysexecutive syndrome that accompanies FXTAS appears to play a significant role, along with the movement disorder, in a significant decline in functional independence, and the development of psychiatric symptomatology.

Among carriers of the *FMR1* premutation who do not have FXTAS, the data suggest that a subset of individuals has mild executive impairment. Whether the cognitive deficits shown by these people indicate preclinical FXTAS, or are neurodevelopmental features of a premutation phenotype, is uncertain, although some imaging data suggest that both are possibilities [89]. Also unclear is whether cognitive problems precede, follow, or are contemporaneous with the onset of tremor and ataxia, and whether these may be associated with neuropathologic and/or neuroradiologic anomalies. Neurophysiologic and neuroimaging data, in conjunction with neuropsychological examination, may help to answer this question.

## Abbreviations

AD: Alzheimer’s disease; EF: executive functioning; ERP: event-related potential; FMR1: fragile X mental retardation 1; FMRP: fragile X mental retardation protein; FX: fragile X; FXS: fragile X syndrome; FXTAS: fragile X-associated tremor/ataxia syndrome; IPS: intraparietal sulcus; IQ: intelligence quotient; MRI: magnetic resonance imaging; WAIS: Wechsler Adult Intelligence Scale.

## Competing interests

The authors declare that they have no competing interests.

## Authors' contributions

All authors contributed to the writing of this review. All authors read and approved the final manuscript.

## References

[B1] Hagerman RJ Leehey M Heinrichs W Tassone F Wilson R Hills J Grigsby J Gage B Hagerman PJ Intention tremor, Parkinsonism and generalized brain atrophy in older male carriers of fragile X Neurology 2001 57 127 130 10.1212/WNL.57.1.127 11445641

[B2] Grigsby J Brega AG Jacquemont S Loesch DZ Leehey MA Goodrich GK Hagerman RJ Epstein J Wilson R Cogswell JB Jardini T Hagerman PJ Impairment in the cognitive functioning of men with Fragile X Tremor-Ataxia Syndrome (FXTAS) J Neurol Sci 2006 248 227 233 10.1016/j.jns.2006.05.016 16780889

[B3] Grigsby J Brega AG Leehey MA Goodrich GK Jacquemont S Loesch DZ Cogswell JB Epstein J Wilson R Jardini T Gould E Bennett RE Hessl D Cohen S Cook K Tassone F Hagerman PJ Hagerman RJ Impairment of executive cognitive functioning in males with fragile X-associated tremor/ataxia syndrome (FXTAS) Mov Disord 2007 22 645 650 10.1002/mds.21359 17266074

[B4] Grigsby J Brega AG Engle K Leehey MA Hagerman RJ Tassone F Hessl D Hagerman PJ Cogswell JB Bennett RE Cook K Hall DA Bounds LS Paulich MJ Reynolds A Cognitive profile of fragile X premutation carriers with and without fragile X-associated tremor/ataxia syndrome Neuropsychology 2008 22 48 60 18211155 10.1037/0894-4105.22.1.48

[B5] Brega AG Goodrich G Bennett RE Hessl D Engle K Leehey MA Bounds LS Paulich MJ Hagerman RJ Hagerman PJ Cogswell JB Tassone F Reynolds A Kooken R Kenny M Grigsby J The primary cognitive deficit among males with fragile X-associated tremor/ataxia syndrome (FXTAS) is a dysexecutive syndrome J Clin Exp Neuropsychol 2008 30 853 869 10.1080/13803390701819044 18608667 PMC4098148

[B6] Fuster JM Executive frontal functions Exp Brain Res 2000 133 66 70 10.1007/s002210000401 10933211

[B7] Quintana J Fuster JM From perception to action: temporal integrative functions of prefrontal and parietal neurons Cereb Cortex 1999 9 213 221 10.1093/cercor/9.3.213 10355901

[B8] Grigsby J Leehey MA Jacquemont S Brunberg JA Hagerman RJ Wilson R Epstein JH Greco CM Tassone F Hagerman PJ Cognitive impairment in a 65-year-old male with fragile X-associated tremor-ataxia syndrome (FXTAS) Cogn Behav Neurol 2006 19 165 171 10.1097/01.wnn.0000213906.57148.01 16957495

[B9] Folstein MF Folstein SE McHugh PR “Mini-mental state”. A practical method for grading the cognitive state of patients for the clinician J Psychatr Res 1975 12 189 198 10.1016/0022-3956(75)90026-6 1202204

[B10] Seritan AL Nguyen DV Tomaszewski F Hinton LW Grigsby J Bourgeois JA Hagerman R Dementia in fragile X-associated tremor/ataxia syndrome (FXTAS): Comparison with Alzheimer’s Disease Am J Med Genet Part B: Neuropsychiatr Genet 2008 147B 1138 1144 10.1002/ajmg.b.30732 PMC2898561 18384046

[B11] Seritan A Cogswell J Grigsby J Cognitive dysfunction in *FMR1* premutation carriers Curr Psychiatr Rev 2012 9 78 84 10.2174/157340013805289635 PMC4304642 25620901

[B12] Brega AG Reynolds A Bennet RE Leehey MA Bounds LS Cogswell JB Hagerman RJ Hagerman PJ Grigsby J Functional status of men with the fragile X premutation, with and without the tremor/ataxia syndrome (FXTAS) Int J Geriatr Psychiat 2009 24 1101 1109 10.1002/gps.2231 PMC4414034 19404994

[B13] Yang JC Chan SH Khan S Schneider A Nanakul R Teichholtz S Niu YQ Seritan A Tassone F Grigsby J Hagerman PJ Hagerman RJ Olichney JM Neural substrates of executive dysfunction in fragile X-associated tremor/ataxia syndrome (FXTAS): a brain potential study Cereb Cortex 2012 23 2657 2666 22918986 10.1093/cercor/bhs251 PMC3792740

[B14] Yang JC Simon C Niu YQ Bogost M Schneider A Tassone F Seritan A Grigsby J Hagerman PJ Hagerman RJ Olichney JM Phenotypes of hypofrontality in older female fragile X premutation carriers Ann Neurol 2013 74 275 283 23686745 10.1002/ana.23933 PMC3906211

[B15] Schmahmann JD Disorders of the cerebellum: ataxia, dysmetria of thought, and the cerebellar cognitive affective syndrome J Neuropsychiatry Clin Neurosci 2004 16 367 378 15377747 10.1176/jnp.16.3.367

[B16] Schmahmann JD Smith EE Eichler FS Filley CM Cerebral white matter: Neuroanatomy, clinical neurology, and neurobehavioral correlates Ann N Y Acad Sci 2008 1142 266 309 10.1196/annals.1444.017 18990132 PMC3753195

[B17] Greco CM Berman RF Martin RM Tassone F Schwartz PH Brunberg JA Grigsby J Hessl D Becker EJ Papazian J Leehey MA Hagerman RJ Hagerman PJ Neuropathology of fragile X-associated tremor-ataxia syndrome (FXTAS) Brain 2006 129 243 255 16332642 10.1093/brain/awh683

[B18] Brunberg JA Jacquemont S Hagerman RJ Berry-Kravis EM Grigsby J Leehey MA Tassone F Brown WT Greco C Hagerman PJ Fragile X premutation carriers: characteristic MR imaging findings in adult males with progressive cerebellar and cognitive dysfunction Am J Neuroradiol 2002 23 1757 1766 12427636 PMC8185834

[B19] Cohen S Masyn K Adams J Hessl D River S Tassone F Brunberg J DeCarli C Zhang L Cogswell J Loesch D Leehey M Grigsby J Hagerman PJ Hagerman R Molecular and imaging correlates of the fragile X-associated tremor ataxia syndrome (FXTAS) Neurology 2006 67 1426 1431 10.1212/01.wnl.0000239837.57475.3a 17060569

[B20] Filley CM Franklin GM Heaton RK Rosenberg NL White matter dementia: clinical disorders and implications Neuropsychiatry Neuropsychol Behav Neurol 1988 1 239 254

[B21] Filley CM The behavioral neurology of cerebral white matter Neurology 1998 50 1535 1540 10.1212/WNL.50.6.1535 9633691

[B22] Tullberg M Fletcher E DeCarli C Mungas D Reed BR Harvey DJ Weiner MW Chui HC Jagust WJ White matter lesions impair frontal lobe function regardless of their location Neurology 2004 63 246 253 10.1212/01.WNL.0000130530.55104.B5 15277616 PMC1893004

[B23] Coffey SM Cook K Tartaglia N Tassone F Nguyen DV Pan R Bronsky HE Yuhas J Borodyanskaya M Grigsby J Doerflinger M Hagerman PJ Hagerman RJ Expanded clinical phenotype of women with the *FMR1* premutation Am J Med Genet A 2008 146A 1009 1016 10.1002/ajmg.a.32060 18348275 PMC2888464

[B24] Rodriguez-Revenga L Madrigal I Pagonabarraga J Xuncla M Badenas C Kulisevsky J Gomez B Mila M Penetrance of *FMR1* premutation associated pathologies in fragile X syndrome families Eur J Hum Genet 2009 17 1359 1362 10.1038/ejhg.2009.51 19367323 PMC2986640

[B25] Adams JS Adams PE Nguyen D Brunberg JA Tassone F Zhang W Koldewyn K Rivera SM Grigsby J Zhang L DeCarli C Hagerman PJ Hagerman RJ Volumetric brain changes in females with fragile X-associated tremor/ataxia syndrome (FXTAS) Neurology 2007 2007 69 851 859 17724287 10.1212/01.wnl.0000269781.10417.7b

[B26] Apartis E Blancher A Meissner WG Guyant-Marechal L Maltete D De Broucker T Legrand AP Bouzenada H Thanh HT Sallansonnet-Froment M Wang A Tison F Roué-Jagot C Sedel F Charles P Whalen S Héron D Thobois S Poisson A Lesca G Ouvrard-Hernandez AM Fraix V Palfi S Habert MO Gaymard B Dussaule JC Pollak P Vidailhet M Durr A Barbot JC FXTAS: new insights and the need for revised diagnostic criteria Neurology 2012 79 1898 1907 10.1212/WNL.0b013e318271f7ff 23077007

[B27] Berry-Kravis E Lewin F Wuu J Leehey M Hagerman R Hagerman P Goetz CG Tremor and ataxia in fragile X premutation carriers: blinded videotape study Ann Neurol 2003 53 616 623 10.1002/ana.10522 12730995

[B28] Berry-Kravis E Abrams L Coffey SM Hall DA Greco C Gane LW Grigsby J Bourgeois J Finucane B Jacquemont S Brunberg JA Zhang L Lin J Tassone F Hagerman PJ Hagerman RJ Leehey MA Fragile X-associated tremor/ataxia syndrome: clinical features, genetics, and testing guidelines Mov Disord 2007 22 2018 2030 10.1002/mds.21493 17618523

[B29] Jacquemont S Hagerman RJ Leehey M Grigsby J Zhang L Brunberg JA Greco C Des Portes V Jardini T Levine R Berry-Kravis E Brown WT Schaeffer S Kissel J Tassone F Hagerman PJ Fragile X premutation tremor/ataxia syndrome: molecular, clinical, and neuroimaging correlates Am J Hum Genet 2003 72 869 878 10.1086/374321 12638084 PMC1180350

[B30] Hagerman RJ Leavitt BR Farzin F Jacquemont S Greco CM Brunberg JA Tassone F Hessl D Harris SW Zhang L Jardini T Gane LW Ferranti J Ruiz L Leehey MA Grigsby J Hagerman PJ Fragile-X-associated tremor/ataxia syndrome (FXTAS) in females with the *FMR1* premutation Am J Hum Genet 2004 74 1051 1056 10.1086/420700 15065016 PMC1181968

[B31] Jacquemont S Hagerman RJ Leehey MA Hall DA Levine RA Brunberg JA Zhang L Jardini T Gane LW Harris SW Herman K Grigsby J Greco CM Berry-Kravis E Tassone F Hagerman PJ Penetrance of the fragile X-associated tremor/ataxia syndrome in a premutation carrier population JAMA 2004 291 460 469 10.1001/jama.291.4.460 14747503

[B32] Yang JC Simon C Niu YQ Bogost M Schneider A Tassone F Seritan A Grigsby J Hagerman PJ Hagerman RJ Olichney JM Phenotypes of hypofrontality in older female fragile X premutation carriers Ann Neurol 2013 20 23933 10.1002/ana.23933 PMC3906211 23686745

[B33] Sterling AM Mailick M Greenberg J Warren SF Brady N Language dysfluencies in females with the *FMR1* premutation Brain Cogn 2013 82 84 89 10.1016/j.bandc.2013.02.009 23523717 PMC3625470

[B34] Tassone F Greco CM Hunsaker MR Seritan AL Berman RF Gane LW Jacquemont S Basuta K Jin LW Hagerman PJ Hagerman RJ Neuropathological, clinical and molecular pathology in female fragile X premutation carriers with and without FXTAS Genes Brain Behav 2012 11 577 585 10.1111/j.1601-183X.2012.00779.x 22463693 PMC3965773

[B35] Adams PE Adams JS Nguyen DV Hessl D Brunberg JA Tassone F Zhang W Koldewyn K Rivera SM Grigsby J Zhang L DeCarli C Hagerman PJ Hagerman RJ Psychological symptoms correlate with reduced hippocampal volume in fragile X premutation carriers Am J Med Genet B Neuropsychiatr Genet 2009 153B 775 785 19908235 10.1002/ajmg.b.31046 PMC2868927

[B36] Hunter JE Rohr JK Sherman SL Co-occurring diagnoses among *FMR1* premutation allele carriers Clin Genet 2010 77 374 381 10.1111/j.1399-0004.2009.01317.x 20059484 PMC3696492

[B37] Kraan C Hocking DR Georgiou-Karistianis N Metcalfe S Archibald A Fielding J Trollor J Bradshaw JL Cohen J Cornish K Impaired response inhibition is associated with self-reported symptoms of depression, anxiety and ADHD in female *FMR1* premutation carriers AJMG 2013 165 41 51 10.1002/ajmg.b.32203 24166828

[B38] Hunter JE Allen EG Abramowitz A Rusin M Leslie M Novak G Hamilton D Shubeck L Charen K Sherman SL Investigation of phenotypes associated with mood and anxiety among male and female fragile X premutation carriers Behav Genet 2008 38 493 502 10.1007/s10519-008-9214-3 18535897 PMC3696488

[B39] Lachiewicz A Dawson D Spiridigliozzi G Cuccaro M Lachiewicz M McConkie-Rosell A Indicators of anxiety and depression in women with the fragile X premutation: assessment of a clinical sample J Intellect Disabil Res 2010 54 597 610 10.1111/j.1365-2788.2010.01290.x 20629912

[B40] Roberts JE Bailey DB Jr Mankowski J Ford A Sideris J Weisenfeld LA Heath TM Golden RN Mood and anxiety disorders in females with the *FMR1* premutation Am J Med Genet B Neuropsychiatr Genet 2009 150B 130 139 18553360 10.1002/ajmg.b.30786

[B41] Nunez PL Srinivasan R Electric Fields of the Brain: the Neurophysics of EEG 2006 2 New York, NY: Oxford University Press 163 166

[B42] Olichney JM Chan S Wong LM Schneider A Seritan A Niese A Yang JC Laird K Teichholtz S Khan S Tassone F Hagerman R Abnormal N400 word repetition effects in fragile X-associated tremor/ataxia syndrome Brain 2010 133 1438 1450 10.1093/brain/awq077 20410144 PMC2859155

[B43] Olichney JM Iragui VJ Salmon DP Riggins BR Morris SK Kutas M Absent event-related potential (ERP) word repetition effects in mild Alzheimer’s disease Clin Neurophysiol 2006 117 1319 1330 10.1016/j.clinph.2006.02.022 16644278 PMC1544116

[B44] Olichney JM Taylor JR Gatherwright J Salmon DP Bressler AJ Kutas M Iragui-Madoz VJ Patients with MCI and N400 or P600 abnormalities are at very high risk for conversion to dementia Neurology 2008 70 1763 1770 18077800 10.1212/01.wnl.0000281689.28759.ab PMC3071795

[B45] Yang JC Simon C Schneider A Hamilton H Hagerman PJ Hagerman RJ JM Abnormal Semantic Processing and Episodic Memory in Females with Fragile X-associated Tremor/Ataxia Syndrome (FXTAS) [abstract] 2013 Hawaii: The 41st International Neuropsychological Society (INS) annual meeting, Feb 2–6

[B46] Grunwald T Beck H Lehnertz K Blümcke I Pezer N Kurthen M Fernández G Van Roost D Heinze HJ Kutas M Elger CE Evidence relating human verbal memory to hippocampal N-methyl-D-aspartate receptors Proc Natl Acad Sci U S A 1999 96 12085 12089 10.1073/pnas.96.21.12085 10518580 PMC18416

[B47] Yang JC Simon C Niu YQ Chen L Seritan A Schneider A Hagerman PJ Hagerman RJ Olichney JM Effects of Memantine on Language/Memory-related Brain Potentials in Fragile X-associated Tremor/Ataxia Syndrome (FXTAS) 2013 Perugia, Italy: The 1st International Conference on FMR1 Premutation: Basic Mechanisms and Clinical Involvement

[B48] Seritan AL Nguyen DV Mu Y Tassone F Bourgeois JA Schneider A Cogswell J Cook K Leehey M Grigsby J Olichney JM Adams P Legg W Zhang L Hagerman PJ Hagerman RJ Memantine for fragile X-associated tremor/ataxia syndrome (FXTAS): a randomized, double-blind, placebo-controlled trial J Clin Psychiatryin press 10.4088/JCP.13m08546 PMC4296896 24345444

[B49] Karmon Y Gadoth N Fragile X associated tremor/ataxia syndrome (FXTAS) with dementia in a female harbouring *FMR1* premutation J Neurol Neurosurg Psychiatry 2008 79 738 739 18487560 10.1136/jnnp.2007.139642

[B50] Schneider A Ballinger E Chavez A Tassone F Hagerman RJ Hessl D Prepulse inhibition in patients with fragile X-associated tremor ataxia syndrome Neurobiol Aging 2012 33 1045 1053 10.1016/j.neurobiolaging.2010.09.002 20961665 PMC3044775

[B51] Cornish Hocking DR Moss SA Kogan CS Selective executive markers of at-risk profiles associated with the fragile X premutation Neurology 2011 77 618 622 10.1212/WNL.0b013e3182299e59 21775729 PMC3159093

[B52] Cornish Kogan CS Li L Turk J Jacquemont S Hagerman RJ Lifespan changes in working memory in fragile X premutation males Brain Cogn 2009 69 551 558 10.1016/j.bandc.2008.11.006 19114290 PMC4158922

[B53] Cornish K Li L Kogan CS Jacquemont S Turk J Dalton A Hagerman RJ Hagerman PJ Age-dependent cognitive changes in carriers of the fragile X syndrome Cortex 2008 44 628 636 10.1016/j.cortex.2006.11.002 18472033 PMC11060834

[B54] Wechsler D WMS-III: Wechsler Memory Scale-Third Edition 1997 San Antonio: Psychological Corporation

[B55] Hashimoto RI Backer KC Tassone F Hagerman RJ Rivera SM An fMRI study of the prefrontal activity during the performance of a working memory task in premutation carriers of the fragile X mental retardation 1 gene with and without fragile X-associated tremor/ataxia syndrome (FXTAS) J Psychiatr Res 2011 45 36 43 10.1016/j.jpsychires.2010.04.030 20537351 PMC2978252

[B56] Hunter JE Abramowitz A Rusin M Sherman SL Is there evidence for neuropsychological and neurobehavioral phenotypes among adults without FXTAS who carry the *FMR1* premutation? A review of current literature Genet Med 2009 11 79 89 19265746 10.1097/GIM.0b013e31818de6ee PMC2652667

[B57] Hunter JE Allen EG Abramowitz A Rusin M Leslie M Novak G Hamilton D Shubeck L Charen K Sherman SL No evidence for a difference in neuropsychological profile among carriers and noncarriers of the *FMR1* premutation in adults under the age of 50 Am J Hum Genet 2008 83 692 702 10.1016/j.ajhg.2008.10.021 19026394 PMC2668066

[B58] Hunter JE Sherman SL Grigsby J Kogan CS Cornish K Capturing the fragile X premutation phenotypes: a collaborative effort across multiple cohorts Neuropsychologia 2012 26 156 164 10.1037/a0026799 PMC3295926 22251309

[B59] Cornish K Kogan C Turk J Manly T James N Mills A Dalton A The emerging fragile X premutation phenotype: evidence from the domain of social cognition Brain Cogn 2005 57 53 60 10.1016/j.bandc.2004.08.020 15629215

[B60] Dorn MB Mazzocco MM Hagerman RJ Behavioral and psychiatric disorders in adult male carriers of fragile X J Am Acad Child Adolesc Psychiatry 1994 33 256 264 10.1097/00004583-199402000-00015 8150798

[B61] Moore CJ Daly EM Schmitz N Tassone F Tysoe C Hagerman RJ Hagerman PJ Morris RG Murphy KC Murphy DGM A neuropsychological investigation of male premutation carriers of fragile X syndrome Neuropsychologia 2004 42 1934 1947 10.1016/j.neuropsychologia.2004.05.002 15381024

[B62] Loesch D Bui QM Grigsby J Butler E Epstein JH Huggins RM Taylor AK Hagerman RJ Effect of the fragile X status categories and the FMRP levels on executive functioning in fragile X males and females Neuropsychology 2003 17 646 657 14599277 10.1037/0894-4105.17.4.646

[B63] Goodrich-Hunsaker NJ Wong LM McLennan Y Srivastava S Tassone F Harvey D Rivera SM Simon TJ Young adult female fragile X premutation carriers show age- and genetically-modulated cognitive impairments Brain Cogn 2011 75 255 260 10.1016/j.bandc.2011.01.001 21295394 PMC3050049

[B64] Goodrich-Hunsaker NJ Wong LM McLennan Y Tassone F Harvey D Rivera SM Simon TJ Adult female fragile X premutation carriers exhibit age- and CGG repeat length-related impairments on an attentionally based enumeration task Front Hum Neurosci 2011 5 14 21808616 10.3389/fnhum.2011.00063 PMC3139190

[B65] Lachiewicz AM Dawson DV Spiridigliozzi GA McConkie-Rosell A Arithmetic difficulties in females with the fragile X premutation Am J Med Genet A 2006 140 665 672 16508954 10.1002/ajmg.a.31082

[B66] Semenza C Bonollo S Polli R Busana C Pignatti R Iuculano T Maria Laverda A Priftis K Murgia A Genetics and mathematics: *FMR1* premutation female carriers Neuropsychologia 2012 50 3757 3763 10.1016/j.neuropsychologia.2012.10.021 23123760

[B67] Kraan CM Hocking DR Bradshaw JL Fielding J Cohen J Georgiou-Karistianis N Cornish KM Neurobehavioural evidence for the involvement of the *FMR1* gene in female carriers of fragile X syndrome Neurosci Biobehav Rev 2013 37 522 547 10.1016/j.neubiorev.2013.01.010 23352653

[B68] Goodrich-Hunsaker NJ Wong LM McLennan Y Tassone F Harvey D Rivera S Simon TJ Enhanced manual and oral motor reaction time in young adult female fragile X premutation carriers J Int Neuropsychol Soc 2011 17 1 5 10.1017/S1355617710001116 21554789 PMC3210929

[B69] Steyaert J Borghgraef M Fryns JP Apparently enhanced visual information processing in female fragile X carriers: preliminary findings Am J Med Genet 1994 51 374 377 10.1002/ajmg.1320510415 7943002

[B70] Chonchaiya W Nguyen DV Au J Campos L Berry-Kravis EM Lohse K Mu Y Utari A Hervey C Wang L Sorensen P Cook K Gane L Tassone F Hagerman RJ Clinical involvement in daughters of men with fragile X-associated tremor ataxia syndrome Clin Genet 2010 78 38 46 10.1111/j.1399-0004.2010.01448.x 20497189 PMC4031089

[B71] Narcisa V Aguilar D Nguyen DV Campos L Brodovsky J White S Adams P Tassone F Hagerman PJ Hagerman RJ A quantitative assessment of tremor and ataxia in female *FMR1* premutation carriers using CATSYS Curr Gerontol Geriatr Res 2011 484713 8 10.1155/2011/484713 PMC3114433 23008705

[B72] Kraan CM Hocking DR Georgiou-Karistianis N Metcalfe SA Archibald AD Fielding J Trollor J Bradshaw JL Cohen J Cornish KM Cognitive-motor interference during postural control indicates at-risk cerebellar profiles in females with the *FMR1* premutation Behav Brain Res 2013 26 00432 00434 10.1016/j.bbr.2013.07.033 23896050

[B73] Bailey DB Jr Raspa M Olmsted M Holiday DB Co-occurring conditions associated with *FMR1* gene variations: findings from a national parent survey Am J Med Genet A 2008 146A 2060 2069 10.1002/ajmg.a.32439 18570292

[B74] Johnston C Eliez S Dyer-Friedman J Hessl D Glaser B Blasey C Taylor A Reiss A Neurobehavioral phenotype in carriers of the fragile X premutation Am J Med Genet 2001 103 314 319 10.1002/ajmg.1561 11746012

[B75] Hunter JE Leslie M Novak G Hamilton D Shubeck L Charen K Abramowitz A Epstein MP Lori A Binder E Cubells JF Sherman SL Depression and anxiety symptoms among women who carry the *FMR1* premutation: impact of raising a child with fragile X syndrome is moderated by CRHR1 polymorphisms Am J Med Genet B Neuropsychiatr Genet 2012 159B 549 559 22573456 10.1002/ajmg.b.32061 PMC3696495

[B76] Seltzer MM Barker ET Greenberg JS Hong J Coe C Almeida D Differential sensitivity to life stress in *FMR1* premutation carrier mothers of children with fragile X syndrome Health Psychol 2012 31 612 622 22149120 10.1037/a0026528 PMC3434309

[B77] Kenna HA Tartter M Hall SS Lightbody AA Nguyen Q De Los Angeles CP Reiss AL Rasgon NL High rates of comorbid depressive and anxiety disorders among women with premutation of the *FMR1* gene Am J Med Genet B Neuropsychiatr Genet 2013 3 32196 10.1002/ajmg.b.32196 PMC5756731 24003006

[B78] Zangenehpour S Cornish KM Chaudhuri A Whole-brain expression analysis of FMRP in adult monkey and its relationship to cognitive deficits in fragile X syndrome Brain Res 2009 1264 76 84 19368811 10.1016/j.brainres.2009.01.059

[B79] Tassone F Hagerman RJ Garcia-Arocena D Khandjian EW Greco CM Hagerman PJ Intranuclear inclusions in neural cells with premutation alleles in fragile X associated tremor/ataxia syndrome J Med Genet 2004 41 e43 10.1136/jmg.2003.012518 15060119 PMC1735735

[B80] Hashimoto R Javan AK Tassone F Hagerman RJ Rivera SM A voxel-based morphometry study of grey matter loss in fragile X-associated tremor/ataxia syndrome Brain 2011 134 863 78 10.1093/brain/awq368 21354978 PMC3044831

[B81] Koldewyn K Hessl D Adams J Tassone F Hagerman PJ Hagerman RJ Rivera SM Reduced hippocampal activation during recall is associated with elevated *FMR1* mRNA and psychiatric symptoms in men with the fragile X premutation Brain Imaging Behav 2008 2 105 116 10.1007/s11682-008-9020-9 19430586 PMC2678852

[B82] Kim SY Hashimoto RI Tassone F Simon TJ Rivera SM Altered neural activity of magnitude estimation processing in adults with the fragile X premutation J Psychiatr Res 2013 47 1909 1916 10.1016/j.jpsychires.2013.08.014 24045061 PMC3880247

[B83] Hagerman P Current gaps in understanding the molecular basis of FXTAS Tremor Other Hyperkinet Mov 2012 2 http://tremorjournal.org/article/view/63 10.7916/D80C4TH0 PMC3379894 23440729

[B84] Tassone F Adams J Berry-Kravis EM Cohen SS Brusco A Leehey MA LI L Hagerman RJ Hagerman PJ CGG repeat length correlates with age of onset of motor signs of the fragile X-associated tremor/ataxia syndrome (FXTAS) Am J Med Genet B Neuropsychiat Genet 2007 144B 566 569 10.1002/ajmg.b.30482 17427188

[B85] Leehey MA Berry-Kravis E Goetz CG Zhang L Hall DA Li L Rice CD Lara R Cogswell J Reynolds A Gane L Jacquemont S Tassone F Grigsby J Hagerman R Hagerman PJ FMR1 CGG repeat length predicts motor dysfunction in premutation carriers Neurology 2008 70 1397 1402 10.1212/01.wnl.0000281692.98200.f5 18057320 PMC2685188

[B86] Bourgeois JB Farzin F Brunberg JA Tassone F Hagerman P Zhang L Hessl D Hagerman R Dementia with mood symptoms in a fragile X premutation carrier with the fragile X-associated tremor/ataxia syndrome: clinical intervention with donepezil and venlafaxine J Neuropsychiatry Clin Neurosci 2006 18 171 177 16720793 10.1176/jnp.2006.18.2.171

[B87] Hagerman RJ Hall DA Coffey S Leehey M Bourgeois J Gould J Zhang L Seritan A Berry-Kravis E Olichney J Miller JW Fong AL Carpenter R Bodine C Gane LW Rainin E Hagerman H Hagerman PJ Treatment of fragile X-associated tremor ataxia syndrome (FXTAS) and related neurological problems Clin Interv Aging 2008 3 251 262 18686748 10.2147/cia.s1794 PMC2546470

[B88] Leehey MA Fragile X-associated tremor/ataxia syndrome: clinical phenotype, diagnosis, and treatment J Investig Med 2009 57 830 836 19574929 10.231/JIM.0b013e3181af59c4 PMC2787702

[B89] Battistella G Niederhauser J Fornari E Hippolyte L Perrin AG Lesca G Forzano F Hagmann P Vingerhoets FJG Draganski B Maeder P Jacquemont S Brain structure in asymptomatic *FMR1* premutation carriers at risk for fragile X-associated tremor/ataxia syndrome Neurobiol Aging 2013 34 1700 1707 10.1016/j.neurobiolaging.2012.12.001 23298734

